# Validation of a hemoglobin A_1c_ model in patients with type 1 and type 2 diabetes and its use to go beyond the averaged relationship of hemoglobin A_1c_ and mean glucose level

**DOI:** 10.1186/s12967-014-0328-5

**Published:** 2014-12-10

**Authors:** Piotr Ladyzynski, Piotr Foltynski, Marianna I Bak, Stanislawa Sabalinska, Janusz Krzymien, Jerzy Kawiak

**Affiliations:** Nalecz Institute of Biocybernetics and Biomedical Engineering, Polish Academy of Sciences, 4 Trojdena street, 02-109 Warsaw, Poland; Clinic and Department of Gastroenterology and Metabolic Diseases, Medical University of Warsaw, 1A Banacha street, 02-097 Warsaw, Poland

**Keywords:** Diabetes mellitus, Glycated hemoglobin A1c, Diabetes management, Glycemic control, Chronic glycemia, Continuous glucose monitoring, Cultivation of erythrocytes in vitro

## Abstract

**Background:**

Glycated hemoglobin A_1c_ (HbA_1c_) has been used as an index of glycemic control in the management, guidance, and clinical trials of diabetic patients for the past 35 years. The aim of this study was to validate the HbA_1c_ model in patients with type 1 and type 2 diabetes and to use it to support interpretation of HbA_1c_ in different clinical situations.

**Methods:**

The HbA_1c_ model was identified in 30 patients (15 with type 1 diabetes and 15 with type 2 diabetes) by estimating the overall glycation rate constant (*k*), based on results of continuous glucose monitoring. The model was validated by assessing its ability to predict HbA_1c_ changes in cultures of erythrocytes *in vitro* and to reproduce results of the A1C-Derived Average Glucose (ADAG) study. The model was used to simulate the influence of different glucose profiles on HbA_1c_.

**Results:**

The mean *k* was equal to 1.296 ± 0.216 × 10^−9^ l mmol^−1^ s^−1^ with no difference between type 1 and type 2 diabetes. The mean coefficient of variation of *k* was equal to 16.7%. The model predicted HbA_1c_ levels *in vitro* with a mean absolute difference less than 0.3% (3.3 mmol/mol). It reproduced the linear relationship of HbA_1c_ and mean glucose levels established in the ADAG study. The simulation experiments demonstrated that during periods of unstable glycemic control, glycemic profiles with the same mean glucose might result in much different HbA_1c_ levels.

**Conclusions:**

Patients with type 1 and type 2 diabetes are characterized by the same mean value of *k*, but there is considerable interindividual variation in the relationship of HbA_1c_ and mean glucose level. Results suggest that reciprocal changes in glycation rate and the life span of erythrocytes exist in a wide range of HbA_1c_ values. Thus, for an average patient with diabetes, no modifications of parameters of the glycation model are required to obtain meaningful HbA_1c_ predictions. Interpreting HbA_1c_ as a measure of the mean glucose is fully justified only in the case of stable glycemia. The model and more frequent tests of HbA_1c_ might be used to decrease ambiguity of interpreting HbA_1c_ in terms of glycemic control.

## Background

Glycated hemoglobin A_1c_ (HbA_1c_) has been used as an index of glycemic control in the management, clinical guidance, and clinical trials of diabetic patients for the past 35 years. In 2009, HbA1c became a diagnostic test for diabetes [1]. In 2008, the multi-center A1C-Derived Average Glucose (ADAG) study was concluded, documenting the linear relationship between HbA_1c_ and mean blood glucose (MBG) [2]. For an average individual, this relationship can be used to report HbA_1c_ as an estimated average glucose level over a period of 3 to 4 months (which is considered to be an approximate life span of erythrocytes) preceding HbA_1c_ test execution. According to the ADAG study assumptions, such an estimate should fall within ±15% of the study-wide calculated level for 90% of the individual patients [2]. However, this average linear relationship cannot be combined with any additional knowledge about the particular patient (e.g., results of HbA_1c_ tests repeated within a short time period or information from a patient that she or he experienced a substantial improvement in glycemic control a few weeks ago) to narrow this 15% uncertainty range. Neither can it be used to study the influence of different glycemia profiles on the HbA_1c_ level. For this purpose, mathematical modeling has been applied, among other methodologies [3-12].

In 2011, Ladyzynski *et al.* demonstrated that it was feasible to approximate the average relationship of HbA_1c_ and glycemia reported in the ADAG study using one of such models [12]. The kinetics of hemoglobin glycation in this model can be characterized by an overall hemoglobin glycation rate constant (*k*). Besides the kinetics of the hemoglobin glycation reaction, the release of erythrocytes to the blood stream from the bone marrow and the elimination of erythrocytes from circulation were taken into account while calculating the average HbA_1c_ level over all equal-aged cohorts of erythrocytes circulating in the vascular system at any given time.

Because the above-mentioned report [12] was based on data from healthy volunteers, it seemed advisable to validate the model using data from patients with diabetes. We expected the mean values of *k* to be similar in patients with type 1 and type 2 diabetes. However, we have not found any data in the literature confirming such an equality of the glycation rate constants in these two groups of patients. In reports available in the literature, the total number of cases studied so far in patients with diabetes is limited, making it difficult to draw conclusions about the mean values and the intersubject variability of *k* in type 1 and type 2 diabetes. Contrarily, many clinical studies reported high variability of HbA_1c_, which could hardly be explained by differences in glycemic control. Taking into consideration the different pathophysiology of type 1 and type 2 diabetes and considering all the factors other than glycemia that might influence the glycation rate (e.g., pH, oxidative stress, enzymatic deglycation, Schiff base inhibitors), the possibility that there are significant differences in formation of HbA_1c_ in these two groups of patients cannot be ruled out.

The aim of the current work was threefold: (1) to estimate and compare the mean *k* and its interindividual variability in patients with type 1 and type 2 diabetes, (2) to validate the ability of the mathematical model to predict HbA_1c_ concentration based on different glucose levels and to reproduce the relationship of HbA_1c_ and glycemia established in the ADAG study, and (3) to simulate different glycemia profiles and their influence on the HbA_1c_ concentration and to use these simulations to support interpretation of HbA_1c_ in different clinical situations.

## Methods

In the first part of the study, an experimental procedure described in detail elsewhere [7,12] was used to estimate *k* and to evaluate the HbA_1c_ model. The procedure consisted of four phases, described below.

### Blood glucose and HbA_1c_ estimation *in vivo*

Glycemia course over a 120-day period was estimated based on CGM using a Guardian RT system (Medtronic Diabetes, Northridge, CA, USA) calibrated at least 4 times a day using capillary glucose measured with an Accu-Chek Go glucometer (Roche Diagnostics, Basel, Switzerland). Glucose concentrations measured with glucometers were rescaled to reflect the whole blood glucose concentrations as if they had been measured with the gold standard glucose analyzer YSI 2300 Stat Plus (Yellow Springs Instruments Inc., Yellow Springs, OH, USA) according to the linear regression reported by Cohen *et al.* [13]. Then the results were multiplied by 1.11, as recommended by the International Federation of Clinical Chemistry and Laboratory Medicine (IFCC) [14], to reflect blood glucose (BG) concentrations in plasma. Based on the DirectNet study, it was assumed that Guardian RT neither underestimates nor overestimates glucose concentration in relation to the calibrating results [15]. In each participant, 3 glucose sensors were applied for 6 days, with an assumed time span of 4 and 2 weeks between application of the first two and the last two sensors, respectively.

Two methods were used to estimate 120-day glycemia profiles. In the first method, we calculated two separate daily glycemia profiles representing working days and weekends by a point-wise averaging of the daily recordings (WW method). Then we connected these profiles repeatedly to obtain the extrapolated 120-day course. In the second method, the rescaled-to-plasma daily profiles were repeatedly copied to build the whole 120-day course, without any intermediate averaging (ID method). Both 120-day profiles were used to identify the individual *k* value for each subject and to evaluate the sensitivity of this estimate on the short-term glycemia variability. The *k* value was also calculated based on an analytical solution of the model under the assumption that BG was equal to the mean value (MBG) for 120 days.

The HbA_1c_ was measured at the end of usage of the last sensor (5 repetitions were done) by applying the cation-exchange HPLC method with a D-10 analyzer (Bio-Rad Laboratories, Hercules, CA, USA). This analyzer measures HbA_1c_ according to the National Glycohemoglobin Standardization Programme (NGSP) as a percentage of the total hemoglobin [16].

### Cultivation of erythrocytes *in vitro*

At the end of glucose monitoring, 30 ml of blood was sampled for cultivation of erythrocytes. The erythrocytes isolated from the blood samples were cultured for up to 5 weeks at 37°C [7,12]. Three glucose levels were maintained in culturing media corresponding to BG of 5.2 mmol/l, 10.5 mmol/l, and 15.7 mmol/l, respectively. Glucose concentrations measured in the medium using the YSI analyzer were divided by 1.06 to account for different water content in the plasma and in the medium [14].

The following procedure was used every day to sustain the presumed constant concentrations of glucose. First, a sample of the medium was taken to measure the glucose concentration before the old medium was replaced by the fresh one. Second, the hemolized erythrocytes were removed together with the old medium from a cell-culture dish, and then the fresh medium containing the desirable glucose concentration was added into the culture. The difference between the glucose concentration at the beginning and at the end of each day was decreasing with time because the number of viable erythrocytes that were able to metabolize glucose was also decreasing. Therefore, the glucose concentration in each culture was not constant but instead was changing, in a sawtooth-like manner, each day. To minimize errors that were made during HbA_1c_ modeling, we interpolated these intraday changes of glucose concentration and used the interpolated values in the model.

A series of preliminary experiments with different glucose concentrations in the medium [7] confirmed that after 14 days of culturing, the molality of glucose in the medium and in the erythrocytes was the same (standard deviation of the absolute relative differences was equal to 3.6%). Glucose content in erythrocytes was not measured after the 14th day of culturing because of a limited volume of samples. Thus, it cannot be ruled out that glucose transport through the walls of the erythrocytes may be affected *in vitro* as a result of changes in the availability of GLUT1, which enable the facilitated diffusion of glucose. However, the influence of such changes on the results must have been limited in the current study because the constant levels of glucose were maintained in the medium.

We also sampled the cultures to measure HbA_1c_ and to estimate the number of viable erythrocytes using Bürker’s chamber [17]. Samples for HbA_1c_ testing were frozen at −80°C until erythrocyte cultivation ended, and then HbA_1c_ was assessed in all samples. To ensure the viability of erythrocytes *in vitro* (or our ability to properly remove nonviable cells and to detect viable cells), in the preliminary tests we used two methods in parallel to estimate the number of the viable cells in the cultures: a microscopic method with Bürker’s chamber and a cytometric method applying the Annexin V binding protocol. In the preliminary tests, described above [7], we conducted six *in vitro* experiments using blood samples from the healthy volunteers. The apoptotic cells were detected (in 5 samples in each experiment) using a FACSCalibur cytometer and CellQuest software (Becton Dickinson, San Diego, CA, USA).

We found a good agreement between the results of the cytometric and the microscopic analyses. The mean difference of the viable erythrocytes count expressed in relation to the erythrocytes’ count at the day of blood sampling for the *in vitro* experiments between the microscopic and the cytometric methods equaled 1.7 ± 2.8% (mean ± SD), p < 0.002 [7]. This result confirmed that we were able to properly distinguish the apoptotic cells from the viable cells. Thus, we used the microscopic method in the main cycle of experiments.

### Estimation of the overall glycation rate constant

The applied HbA_1c_ model assumes that HbA_1c_ level depends on three main processes: the kinetics of hemoglobin glycation, the release of the reticulocytes from bone marrow and the elimination of erythrocytes from circulation. The kinetics of hemoglobin glycation in the equal-aged cohort of erythrocytes was modeled with a simple differential equation [12]:

$$ \frac{d\kern0.5em HbA}{dt}=-k \times HbA \times BG $$

where *HbA* denotes concentration of non-glycated hemoglobin and *t* is time.

The most important assumptions of the model are as follows: (1) the life span of erythrocytes is constant and equal to 120 days, (2) the turnover of erythrocytes is constant, (3) HbA_1c_ concentration in the newly generated reticulocytes is equal to zero [4,18], (4) erythrocytes are eliminated in chronological order (“the oldest” ones are eliminated first) [7], and (5) the influence of the spleen-facilitated vesiculation on HbA_1c_ is negligible [7,10]. The influence of these assumptions on the modeled HbA_1c_ was assessed earlier [7].

Based on these assumptions, a hemoglobin mass balance equation was utilized to calculate HbA_1c_ level in the equal-aged cohort of erythrocytes at any particular point in time, depending on BG. These calculations were performed in parallel in 120 cohorts of erythrocytes of different ages (ranging from 1 day to 120 days), and then the results were averaged over all the cohorts to obtain the modeled HbA_1c_ level that corresponds to the measured HbA_1c_ level. To estimate a value of *k*, calculations were repeated with iteratively modified *k* until the absolute difference between the calculated and the measured HbA_1c_ dropped below 0.046% (0.05 mmol/mol). To avoid overestimation of *k*, the calculations were performed using the unbiased IFCC-aligned HbA_1c_ levels that were obtained from the NGSP-aligned ones according to the linear equation recommended by the IFCC [16]. In this manuscript, HbA_1c_ concentrations are reported according to both scales, with the NGSP-aligned values expressed in percentages of the total hemoglobin (%), followed by the IFCC-aligned values in millimoles of HbA_1c_ per mol of the total hemoglobin (mmol/mol) given in parentheses.

The HbA_1c_ model has been described in detail elsewhere and it has been proven to be capable of predicting HbA_1c_ levels in nondiabetic individuals [7,12]. In one of these reports [12], an analytical solution of the model was presented under an assumption of a constant glycemia throughout the entire life span of erythrocytes (HbA1c is NGSP-aligned and LS stands for the life span of erythrocytes in the equation below):

$$ Hb{A}_{1c}\left(\%\right)=91.5\times \left(1-\frac{1-{e}^{-k\times LS\times MBG}}{k\times LS\times MBG}\right)+2.15 $$

This equation was used to calculate values of *k* for all the study participants, based on their individual MBG values, and to compare them with values of *k* estimated numerically, based on extrapolated continuous glycemia courses obtained using the WW and ID methods.

The most important simplification of the model was related to the assumed constant life span of erythrocytes equal to 120 days. To show an influence of this assumption on the estimated values of *k* we also identified the model (i.e., estimated values of *k* for all the study participants) for alternative values of the life span equal to 60, 80, 100, 140 and 160 days.

### Assessment of the model performance based on *in vitro* data

It was assumed that hemoglobin glycation obeys the same kinetics *in vivo* as it does *in vitro* [6]. The models with *k* individualized for each subject were used to predict HbA_1c_ changes assuming 4 different mechanisms of erythrocyte apoptosis during the *in vitro* cultivation: the chronological loss of cohorts, the uniform loss of erythrocytes from all cohorts, the combination of these two mechanisms, or the counter-chronological loss of erythrocytes (with the “youngest” erythrocytes being eliminated first). The percentage of the modeled erythrocytes that were left each day in the simulated cultures reflected the percentage of the viable erythrocytes measured in the real cultures. No new erythrocytes were added in the model to reflect the real-life conditions. A detailed description of the *in vitro* modeling was reported earlier [7,12]. The mean difference (MD) and the mean absolute difference (MAD) of the measured and the predicted HbA_1c_ levels were used to assess the model’s performance.

### Modeling a relationship of HbA_1c_ and blood glucose levels

In the second part of the study, the mean *k* and its intersubject variability were estimated for patients with type 1 and type 2 diabetes. The mean *k* was then used to model the linear relationship of HbA_1c_ and MBG. The obtained linear function was compared with the experimental one that was reported in the ADAG study [2]. However, the ADAG study reported a regression line with HbA_1c_ as the independent variable, whereas the model-generated results should be compared with the line calculated with MBG as the independent variable (i.e., the one minimizing the prediction error of HbA_1c_ based on MBG). In both cases, the correlation coefficient is the same but the slopes and intercepts of regression lines differ.

We had no access to the raw data from the ADAG population. Therefore, we used the published summary statistics of the ADAG study to build a statistical model of the ADAG population and to draw a sample of 10,000 pairs of HbA_1c_ and MBG from this population with the Monte Carlo technique using the OpenBUGS 3.2.1 system [19]. Based on these simulated data, a regression line of HbA_1c_*vs.* MBG was determined and compared with the relationship obtained using the HbA_1c_ model.

In the third part of the study, the model with the mean *k* was used to simulate the influence of different 120-day-long glucose profiles on HbA_1c_, assuming a 120-day life span of erythrocytes. Additionally, the model with the mean *k* value was used to predict steady-state HbA_1c_ concentrations for constant MBG values in case of shortening (to 60, 80, and 100 days) and lengthening (to 140 and 160 days) of the survival of erythrocytes.

### Participants

The study group consisted of 30 sequentially enrolled non-Hispanic white adults including 15 patients with type 1 diabetes and 15 with type 2 diabetes. Exclusion criteria were proliferative retinopathy or maculopathy requiring treatment, renal impairment (creatinine higher than 177 μmol/l), heart failure (class III or IV, according to NYHA, or cardiac infarction within past 3 months), and mental impairment.

Baseline characteristics of the study group are presented in Table 1. All of the subjects had stable metabolic control prior to the enrollment and a regular lifestyle as confirmed by the results of an interview. The study adhered to the Declaration of Helsinki, the subjects provided informed written consent, and the local ethical committee approved the study protocol.

**Table 1:** **Baseline characteristics of the study group**

	**Type 1**	**Type 2**	**All**
n	15	15	30
Sex (% female)	8 (53)	7 (47)	15 (50)
Age (years)	36.4 ± 15.5	57.5 ± 15.8	47.0 ± 18.8
Duration of diabetes (years)	11.8 ± 11.3	10.1 ± 10.7	10.9 ± 10.8
BMI (kg/m^2^)	23.9 ± 5.6	29.1 ± 5.9	26.5 ± 6.2
C-peptide (nmol/l)	0.11 ± 0.17	1.10 ± 0.63	0.61 ± 0.68
Hemoglobin (g/l)	141 ± 11	139 ± 9	140 ± 10
Hematocrit (%)	41.1 ± 3.1	41.1 ± 2.7	41.1 ± 2.8
RBC (× 10^12^/l)	4.74 ± 0.38	4.65 ± 0.33	4.69 ± 0.35
WBC (× 10^9^/l)	6.78 ± 1.75	7.40 ± 1.78	7.09 ± 1.76
Fe (μmol/l)	17.9 ± 8.6	16.6 ± 3.8	17.2 ± 6.6
Creatinine (μmol/l)	76 ± 27	68 ± 14	72 ± 22

Data are mean ± SD.

BMI, body mass index; RBC, erythrocyte count; WBC, leukocyte count.

### Statistical analysis

Normality of distribution of all variables was confirmed using the Shapiro-Wilk W test. Thus, the analysis of variance (ANOVA) or t-test was applied to analyze the data, using Statistica ver. 7.1 (StatSoft Inc., Tulsa, OK, USA). All results are presented as mean ± SD unless otherwise indicated. A *p*-value below 0.05 was considered statistically significant.

## Results

### Mean value and interindividual variability of the overall glycation rate constant

Table 2 presents the results of *in vivo* monitoring of the metabolic control. The mean HbA_1c_ was higher by 0.7% (7.4 mmol/mol) in patients with type 1 diabetes compared to those with type 2 diabetes (p = 0.07). This difference corresponded with a difference of MBG equal to 1.2 mmol/l (p = 0.02) and 1.3 mmol/l (p = 0.02), extrapolated using the WW and ID methods, respectively. The mean values of the resulting glycemia profiles were not different, regardless of the extrapolation method that was used. However, the mean standard deviation was higher when the ID method was used (p = 0.0000001, 0.000009, and 0.0000001 in patients with type 1 and type 2 diabetes and in the whole study group, respectively).

**Table 2:** **Results of** ***in vivo*** **monitoring of the metabolic control**

	**Type 1 diabetes**	**Type 2 diabetes**	**All participants**
HbA_1c_ (%)	7.3 ± 1.1 (5.3, 9.2)	6.6 ± 0.8 (5.3, 8.4)	7.0 ± 1.0 (5.3, 9.2)
HbA_1c_ (mmol/mol)	57 ± 13 (34, 77)	49 ± 9 (34, 68)	52 ± 11 (34, 77)
Period covered with CGM sensors (days)	17.4 ± 4.3 (10.2, 29.6)	16.2 ± 2.2 (10.6, 18.9)	16.8 ± 3.4 (10.2, 29.6)
Time gap between sensor 1 and 2 (days)	26.6 ± 5.9 (18, 43)	29.3 ± 9.2 (19, 50)	28.0 ± 7.7 (18, 50)
Time gap between sensor 2 and 3 (days)	15.7 ± 6.2 (6, 29)	13.8 ± 8.1 (4, 29)	14.7 ± 7.1 (4, 29)
MBG according to WW method (mmol/l)	8.7 ± 1.6 (6.5, 12.4)	7.5 ± 1.0 (6.1, 9.5)	8.1 ± 1.5 (6.1, 12.4)
MBG according to ID method (mmol/l)	8.7 ± 1.6 (6.4, 12.6)	7.5 ± 1.0(6.1, 9.3)	8.1 ± 1.5 (6.1, 12.6)
SD of BG according to WW method (mmol/l)	1.1 ± 0.3 (0.6, 1.4)	1.2 ± 0.7 (0.3, 3.2)	1.2 ± 0.6 (0.3, 3.2)
SD of BG according to ID method (mmol/l)	2.9 ± 0.7 (1.5, 3.9)	2.1 ± 1.0 (1.1, 4.3)	2.5 ± 0.9 (1.1, 4.3)

Data are mean ± SD (range).

HbA_1c_, hemoglobin A_1c_ concentration; CGM, continuous glucose monitoring; MBG, mean blood glucose concentration; SD, standard deviation; BG, blood glucose concentration; WW method, 120-day glycemia was extrapolated based on two daily profiles for working days and for weekend obtained using the point-wise averaging; ID method, 120-day glycemia was extrapolated based on the glucose data with no averaging.

Table 3 shows a summary of statistics of values of *k* estimated individually for each study participant based on her or his 120-day-long glucose profile extrapolated using the WW and ID methods, as well as values of *k* calculated using an analytical solution of the model assuming a constant glycemia in the full 120-day period. In spite of differences in the glycemic variability, values of *k* estimated using both extrapolation methods and those calculated analytically were almost identical (p = 0.98), regardless of the type of diabetes (p = 0.82), as revealed by ANOVA. The *k* values calculated analytically based on the constant MBG were higher than those iterated using the WW glucose data in 25 out of 30 patients (p = 0.006). However, the mean relative absolute differences were as little as 0.7% ±0.6% and 1.3% ±1.2% and 1.0% ±1.0% in patients with type1 diabetes, patients with type 2 diabetes, and in the whole study group, respectively. In the case of *k* values estimated according to the WW and ID methods, these differences were equal to 0.7% ±1.0% and 1.7% ± 2.1% and 1.2% ±1.7%. Because of the absence of considerable differences in the average values of *k* estimated using three methods, the values of *k* estimated on the basis of the WW method are reported in the remaining part of this work.

**Table 3:** **Results of estimation of the overall hemoglobin glycation rate constant (** ***k*** **)**

	**Type 1 diabetes**	**Type 2 diabetes**	**All participants**
	**× 10** ^**−9**^ **(l mmol** ^**−1**^ **s** ^**−1**^ **)**		
*k* based on BG according to WW method	1.294 ± 0.216 (0.964, 1.794)	1.298 ± 0.223 (1.016, 1.798)	1.296 ± 0.216 (0.964, 1.798)
*k* based on BG according to ID method	1.292 ± 0.222 (0.965, 1.794)	1.307 ± 0.220 (1.008, 1.787)	1.300 ± 0.217 (0.965, 1.794)
*k* calculated analytically based on MBG	1.300 ± 0.218 (0.977, 1.799)	1.313 ± 0.224 (1.024, 1.798)	1.306 ± 0.217 (0.977, 1.799)
Difference of *k* based on ID and WW methods	−0.002 ± 0.015 (−0.037, 0.035)	0.009 ± 0.031 (−0.033, 0.102)	0.003 ± 0.025 (−0.037, 0.102)
Difference of *k* calculated analytically and the one estimated using WW method	0.006 ± 0.010 (−0.009, 0.028)	0.014 ± 0.017 (−0.013, 0.050)	0.010 ± 0.014 (−0.013, 0.050)
Difference of *k* calculated analytically and the one estimated using ID method	0.008 ± 0.013 (−0.010, 0.036)	0.005 ± 0.033 (−0.100, 0.040)	0.007 ± 0.025 (−0.100, 0.040)

Data are mean ± SD (range).

*k*, overall hemoglobin glycation rate constant; BG, blood glucose concentration; WW method, 120-day glycemia was extrapolated based on two daily profiles for working days and for weekend obtained using the point-wise averaging; ID method, 120-day glycemia was extrapolated based on the glucose data with no averaging; MBG, mean blood glucose concentration.

**Figure 1:**
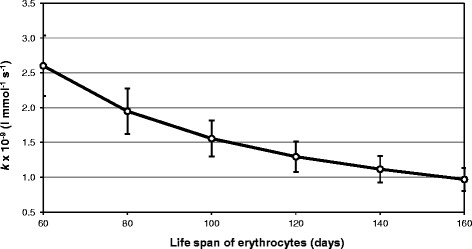
**The overall glycation rate constant (** ***k*** **) estimated for different life spans of erythrocytes.** The mean ± SD of *k* is presented for the life span of erythrocytes ranging from 60 to 160 days.

Figure 1 demonstrates the mean values ± SD of *k* estimated for the life span of erythrocytes ranging from 60 to 160 days. This figure shows that the shorter life span of erythrocytes is assumed to be, the higher the glycation rate must be to adjust the model to the glucose and HbA_1c_ measured in the study participants.

In spite of better metabolic control in patients with type 2 diabetes, the mean *k* values were identical in type 1 and type 2 patients (p = 0.96). The variance of *k* representing the intersubject variability was similar in both subgroups (p = 0.45). The coefficient of variation (CV) of *k* in patients with type 1 diabetes was equal to 16.7%, in those with type 2 diabetes, 17.2%; and in the whole study group, 16.7%.

### Measured and predicted HbA_1c_ concentration *in vitro*

**Figure 2:**
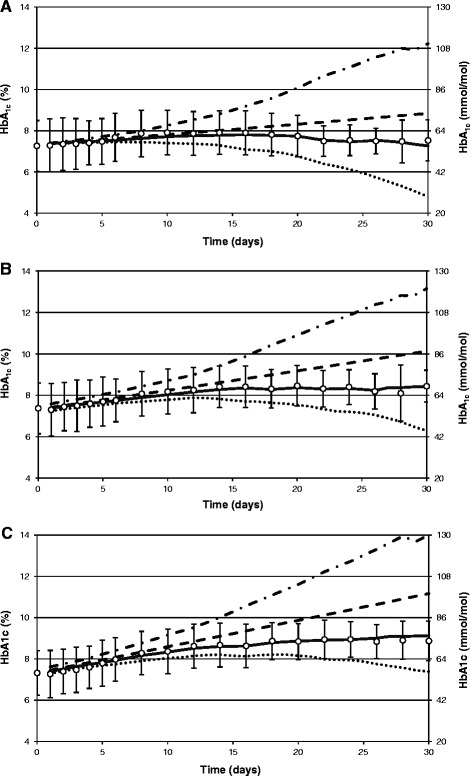
**The measured and the predicted HbA**_**1c**_ **during** ***in vitro*** **cultivation of erythrocytes from patients with type 1 diabetes.** The measured (mean ± SD) HbA_1c_ levels are shown as white circles with error bars and the mean predicted HbA_1c_ levels are shown as: dash-dot lines, dashed line, dotted line and solid line for the counter-chronological, the uniform, the chronological and the combined (i.e. containing the chronological and the uniform component) loss of erythrocytes from the culturing medium, respectively. Glucose concentration is equal to: **(A)** 5.2 mmol/l; **(B)** 10.5 mmol/l; **(C)** 15.7 mmol/l.

**Figure 3:**
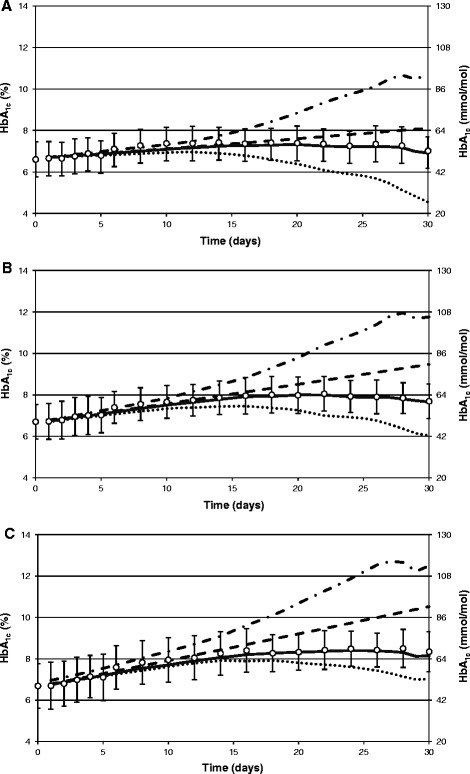
**The measured and the predicted HbA**_**1c**_ **during** ***in vitro*** **cultivation of erythrocytes from patients with type 2 diabetes.** The measured (mean ± SD) HbA_1c_ levels are shown as white circles with error bars and the mean predicted HbA_1c_ levels are shown as: dash-dot lines, dashed line, dotted line and solid line for the counter-chronological, the uniform, the chronological and the combined (i.e. containing the chronological and the uniform component) loss of erythrocytes from the culturing medium, respectively. Glucose concentration is equal to: **(A)** 5.2 mmol/l; **(B)** 10.5 mmol/l; **(C)** 15.7 mmol/l.

Figures 2 and 3 present the measured HbA_1c_ and mean courses of the predicted HbA_1c_ during cultivation of the erythrocytes of patients with type 1 and type 2 diabetes, respectively. Figures 2 and 3 indicates that HbA_1c_ is strongly overestimated when the assumption that erythrocytes are removed counter-chronologically is made.

Overestimation of the real HbA_1c_ concentrations under this assumption can be explained in the following way. At the beginning of culturing of the erythrocytes, there is a mixture of erythrocytes in the culture aged from 0 to 119 days. The *in vitro* simulation starts with the calculated HbA_1c_ levels existing in each equal-aged cohort of erythrocytes at the last day of the glucose monitoring *in vivo*. This means that the content of HbA_1c_ in erythrocytes aged 0 days is almost zero while the content of HbA_1c_ in erythrocytes aged 119 days is almost two times higher (in the case of a stable mean glycemia *in vivo*) than the average content, which is equal to the measured HbA_1c_.

During culturing of erythrocytes *in vitro*, there are two processes that can change the average HbA_1c_: hemoglobin glycation and the removal of nonviable erythrocytes. The first of these processes leads to an increase in the amount of the HbA_1c_ in each viable erythrocyte remaining in the culture. If it were the only process, then the average HbA_1c_*in vitro* would always increase with time. However, the second process may lead to an increase, to stabilization, or to a decrease in the average HbA_1c_, depending on the way in which erythrocytes are eliminated from the culture. If erythrocytes are eliminated in the counter-chronological way, then, in general, the content of HbA_1c_ in erythrocytes being removed is lower than the average content of HbA_1c_ in erythrocytes remaining in the culture, which leads to an increase in the average HbA_1c_. Therefore, under the assumption of the counter-chronological removal of erythrocytes, both above-mentioned processes work together to increase the average HbA_1c_. This assumption seems to be the least realistic because it implies that the oldest erythrocytes can live *in vitro* longer than *in vivo*. That counter-chronological removal of erythrocytes is not realistic is confirmed by the fact that the simulated courses of HbA_1c_ under this assumption are the most different from the real measured courses of HbA_1c_ changes.

It is also noteworthy that differences between the simulated HbA_1c_ concentrations on each particular day for cultures with different glucose concentrations in the case of the counter-chronological removal of the erythrocytes are similar (or even higher) than in the case of the measured differences. For example, in patients with type 1 diabetes, on the 14th day of culturing, differences for the simulated HbA_1c_ concentration are equal to 0.61% between cultures containing 5.2 and 10.5 mmol/l of glucose, and are equal to 0.58% between cultures containing 10.5 and 15.7 mmol/l of glucose. In comparison, for the measured differences, the respective values are equal to 0.53% and 0.25%.

A chronological or uniform elimination of erythrocytes leads to more accurate predictions. The best predictions are obtained assuming the combined loss of erythrocytes containing chronological and uniform components (Table 4). In the whole study group, MDs were similar in cultures with different glucose concentrations (p = 0.29), whereas MADs tended to increase with an increase of glucose level in the culturing medium (p = 0.0000001). However, both indices were very small, demonstrating that the combined apoptosis made it possible to equalize the measured and the predicted mean HbA_1c_ concentrations.

**Table 4:** **Mean difference and mean absolute difference of HbA**_**1c**_ **measured during the** ***in vitro*** **experiments and predicted by the model**

**Glucose concentration**	**Type 1 diabetes**	**Type 2 diabetes**	**All participants**
(mmol/l)	Mean difference (%)*		
5.2	−0.07 ± 0.09	−0.13 ± 0.10	−0.10 ± 0.09
10.5	−0.05 ± 0.08	−0.07 ± 0.08	−0.06 ± 0.07
15.7	−0.00 ± 0.09	−0.13 ± 0.11	−0.06 ± 0.09
	Mean absolute difference (%)*		
5.2	0.15 ± 0.09	0.19 ± 0.08	0.17 ± 0.08
10.5	0.15 ± 0.05	0.22 ± 0.10	0.18 ± 0.06
15.7	0.32 ± 0.06	0.28 ± 0.10	0.30 ± 0.07

Data are mean ± SD.

^*^Mean difference and mean absolute difference in (mmol/mol) can be obtained by multiplying values in (%) by 10.93.

The presented parameters were calculated under the assumption that erythrocytes were eliminated from cultures in combination of the chronological and uniform modes.

### Linear relationship of HbA_1c_ and MBG

The linear relationship of MBG as a function of HbA_1c_ reported in the ADAG study was as follows [2]:

$$ MBG\left( mmol/l\right)=1.5944\times Hb{A}_{1c}\left(\%\right)\hbox{--}\ 2.5944 $$

We obtained almost the same parameters of the regression line using the simulated ADAG population:

$$ MBG\left( mmol/l\right)=1.589\times Hb{A}_{1c}\left(\%\right)\hbox{--}\ 2.559 $$

This equation corresponds to the following relationship when MBG is considered the independent variable:

$$ Hb{A}_{1c}\left(\%\right)=0.5319\times MBG\left( mmol/l\right)+2.419 $$

The relationship obtained using the model was as follows:

$$ Hb{A}_{1c}\left(\%\right)=0.5547\times MBG\left( mmol/l\right)+2.4624 $$

**Figure 4:**
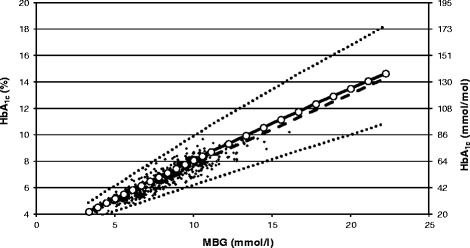
**Relationship of HbA**_**1c**_ **and the mean blood glucose level.** Regression line (dashed line) of HbA_1c_ as a function of the mean blood glucose (MBG) obtained basing on 10,000 samples (the first exemplary 500 sampled data points are shown as black diamonds) drawn from the ADAG study population [2] (*HbA*_*1c*_ *(%) = 0.5319 × MBG(mmol/L) + 2.419*) in comparison with the linear regression obtained based on 25 pairs of MBG and corresponding HbA_1c_ values (white circles) simulated using the model of hemoglobin glycation, assuming the overall glycation rate constant (*k*) of 1.296 × 10^−9^ l mmol^−1^ s^−1^ and a constant life span of erythrocytes of 120 days (*HbA*_*1c*_ *(%) = 0.5547 × MBG(mmol/L) + 2.4624*). The dotted lines were calculated using the model with *k* at the boundaries of a 95% confidence interval (i.e. mean *k* ±1.96 *×* SD).

Figure 4 presents a comparison of the last two linear functions. The model overestimates HbA_1c_ for the given MBG. However, the mean difference over all 10,000 simulated cases is as little as 0.23% ±0.05% (2.5 ± 0.5 mmol/mol). In the range of MBG from 4 to 20 mmol/l, the mean difference of HbA_1c_ estimates is equal to 0.33% (3.6 mmol/mol).

### Changes in HbA_1c_ level in response to different simulated glycemic profiles

**Figure 5:**
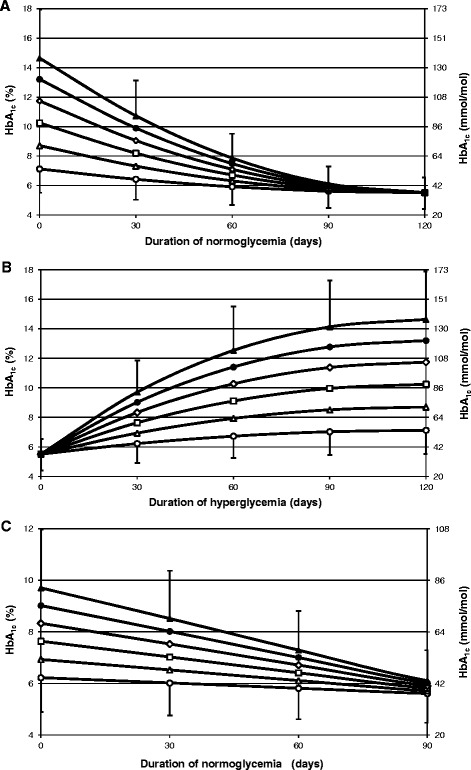
**Changes of HbA**_**1c**_ **level in response to different simulated glycemic profiles. (A)** HbA_1c_ in response to a step improvement of glycemia to 5.6 mmol/l in day zero; **(B)** HbA_1c_ in response to a step deterioration of glycemia from 5.6 mmol/l in day zero, **(C)** HbA_1c_ in response to hyperglycemia lasting for 30 days that ends in day zero (before day −30 and after day zero the glucose level is equal to 5.6 mmol/l). The following hyperglycemia levels were used: 8.3 mmol/l (white circles), 11.1 mmol/l (white triangles), 13.9 mmol/l (white squares), 16.7 mmol/l (white diamonds), 19.4 mmol/l (black circles) and 22.2 mmol/l (black triangles). For two utmost lines error bars were calculated using the model with *k* at the boundaries of a 95% confidence interval (i.e. mean *k* ±1.96 *×* SD).

Figure 5A–C show simulations of different glycemia profiles and their influence on HbA_1c_ level. Figure 5A presents HbA_1c_ changes in response to the step improvement of the glycemia depending on the magnitude of improvement (6 hyperglycemia levels are simulated that drop to the normoglycemia of 5.6 mmol/l) and the duration of this improvement preceding HbA_1c_ test execution. Similarly, Figure 5B shows HbA_1c_ in response to the step deterioration of the glycemia. Based on these figures it can be observed, for example, that 60 days after a drop of BG from 22.2 mmol/l to 5.6 mmol/l, HbA_1c_ is equal to 7.9% (62 mmol/mol), whereas 60 days after a rise in BG from 5.6 mmol/l to 22.2 mmol/l, HbA_1c_ is equal to 12.5% (113 mmol/mol), despite the same average 120-day glycemia in both cases. Using Figure 5A, based on two HbA_1c_ tests equal to 9.9% (85 mmol/mol) and 7.5% (58 mmol/mol) performed 30 days one after another and knowing that MBG was equal to 5.6 mmol/l in both cases, one can predict that the patient experienced a sudden drop of glycemia from 19.4 mmol/l about 30 days before the first HbA_1c_ test was executed.

Figure 5C illustrates the reaction of HbA_1c_ to the temporal (rectangular shape) deterioration of BG lasting for 30 days, depending on the magnitude of this deterioration and a time period between the end of the hyperglycemia and execution of the HbA_1c_ test. For example, in the case of the glycemia deterioration from 5.6 to 22.2 mmol/l lasting for 30 days, HbA_1c_ varies from 9.7% (82.6 mmol/mol) to 6.1% (43.3 mmol/mol), depending on the time span between the end of the hyperglycemia and the day of HbA_1c_ test execution.

**Figure 6:**
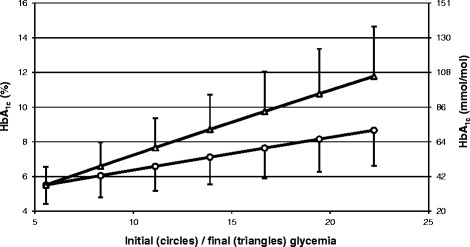
**HbA**_**1c**_ **in response to a ramp improvement (circles) and ramp deterioration (triangles) of glycemia.** The improvement or deterioration of glycemia last for 120-day. In case of the improvement the initial glycemia is equal to: 8.3 mmol/l (white circles), 11.1 mmol/l (white triangles), 13.9 mmol/l (white squares), 16.7 mmol/l (white diamonds), 19.4 mmol/l (black circles) and 22.2 mmol/l (black triangles). The same hyperglycemic levels are used as the final glycemia in case of the ramp of deterioration of the glycemic control. In case of the improvement final glycemia is equal to 5.6 mmol/l and in case of the deterioration the initial glycemia is equal to 5.6 mmol/l. For two utmost lines error bars were calculated using the model with *k* at the boundaries of a 95% confidence interval (i.e. mean *k* ±1.96 *×* SD).

Figure 6 demonstrates changes of HbA_1c_ in response to the ramp improvement and the ramp deterioration of the glycemia lasting for 120 days, depending on the slope of the ramp. For example, for the linear improvement of the glycemia from 22.2 mmol/l to 5.6 mmol/l, HbA_1c_ concentration is equal to 8.7% (71 mmol/mol) and for the deterioration – it is equal to 11.8% (105 mmol/mol). The MBG concentration is the same in both cases, as it was in the examples illustrated in Figures 5A and B.

It should be mentioned that if we assumed an erythrocyte life span different than 120 days, then the HbA_1c_ courses presented in Figures 5A-C would have the same shapes but the time scale would be different. For example, for a life span of 100 days, a new steady-state HbA_1c_ after a step improvement or deterioration of the glycemia would be achieved after 100 days, not 120 days.

**Figure 7:**
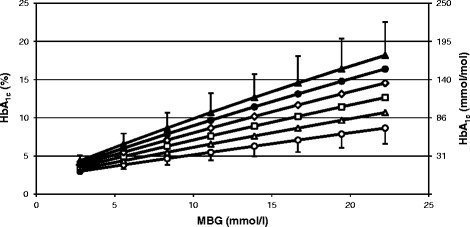
**HbA**_**1c**_ **in response to constant MBG maintained during the whole life span of erythrocytes.** In each simulation the model with *k* equal to 1.296 × 10^−9^ l mmol^−1^ s^−1^ was applied and the following life spans of erythrocytes were used: 60 days (white circles), 80 days (white triangles), 100 days (white squares),120 days (white diamonds), 140 days (black circles), 160 days (black triangles). For two utmost lines error bars were calculated using the model with *k* at the boundaries of a 95% confidence interval (i.e. mean *k* ±1.96 *×* SD).

Figure 7 illustrates an effect of shortening or lengthening of the life span of erythrocytes on HbA_1c_ in a patient having the glycation rate constant equal to the mean value estimated in 30 study participants for the life span of 120 days. Such a shortened life span occurs, for example, in hemolytic or sickle cell anemia and the lengthened life span can be observed in thalassemia.

**Figure 8:**
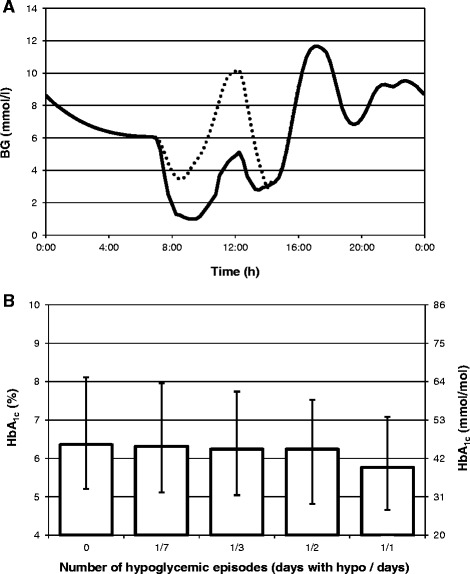
**Changes of HbA**_**1c**_ **level in response to hypoglycemia. (A)** two daily glucose profiles without (dotted line) and with (solid line) an episode of hypoglycemia that were used to compose 120-day glycemia courses with different frequency of hypoglycemic episodes; **(B)** HbA_1c_ in response to 120-day glycemia courses composed of daily profiles shown in panel **(A)** containing the hypoglycemic profile with the following frequencies: never (0), weekly (1/7), every third day (1/3), every second day (1/2), everyday (1/1).

The last simulation assessed whether HbA_1c_ concentration was a sensitive indicator of hypoglycemia or, in general, of short-term glucose variability. For this purpose two daily glucose profiles were prepared using AIDA [20], a model-based online simulator of a patient with type 1 diabetes. One of these profiles includes an episode of severe hypoglycemia (Figure 8A). Both profiles were applied to compose 120-day glycemia courses with variable frequency of the daily hypoglycemic profiles (from “none” to “everyday”), which were used to model HbA_1c_. As shown Figure 8B, for a patient with a mean value of *k*, HbA_1c_ is equal to 6.4% (46 mmol/mol) when no hypoglycemia occurs, and it is equal to 5.8% (40 mmol/mol) when hypoglycemia episodes occur every day. We calculated that there is still a 29% chance that the HbA_1c_ level is higher in a patient experiencing hypoglycemia every day than in a patient who does not experience hypoglycemia at all, because of the intersubject variability of the hemoglobin glycation rate.

## Discussion

The mean overall glycation rate constant values identified using 120-day glycemia courses that were extrapolated based on the CGM data in patients with type 1 and type 2 diabetes support the notion of the same mean rate of hemoglobin glycation in these two groups of diabetic patients. The mean *k* reported in this study is just 3.1% higher than the mean *k* obtained in 10 healthy volunteers, estimated earlier using the same methodology [12], and 2.0% higher than the one that we calculated based on the results of the 3-month CGM data for 22 patients with diabetes and for 3 nondiabetic subjects presented by Nathan *et al.* [21]. The mean *k* reported here is also in a good agreement with previously reported values estimated on the basis of a limited number of samples utilizing *in vivo* or *in vitro* experiments, when methodological differences are accounted for [3,4,6,7,18].

The mean *k* values were only marginally different for three methods of the long-term glycemia extrapolation that were used. This result confirms the data reported in the clinical studies showing that HbA_1c_ is sensitive to MBG level but not to glycemic short-term variations [2,21-23] because the MBG levels used in these three methods were very similar to each other, whereas the glucose variability measures were significantly different.

The life span of erythrocytes shorter or longer than the assumed 120-day would not change the conclusion that the mean *k* is similar in patients with type 1 and type 2 diabetes, provided that the mean erythrocyte survival was similar in these two groups. However, the mean value of *k* would be affected if the erythrocyte life span was different than 120 days.

The interindividual CV of *k* was similar regardless of the type of diabetes, but it was higher than the value reported earlier in healthy volunteers [12] (p = 0.025). The obtained CV is high, but it cannot be automatically attributed in full to variability of glycation rate. Ladyzynski *et al.* [12] demonstrated that a major part of variability of *k* in healthy volunteers could be explained by random errors of HbA_1c_ and glycemia measurements and, more importantly, by a heterogeneity of erythrocyte life span [12].

Furne *et al*. [24] estimated that by using the end-alveolar carbon monoxide technique, the standard deviation of the life span of erythrocytes in healthy subjects was equal to 23 days. This value is more than enough to explain the variability of *k* obtained in the current study and to justify the resulting differences in HbA_1c_ corresponding to a given glucose concentration, which can be observed in Figure 4, where, for example, at an MBG equal to 15.5 mmol/l, HbA_1c_ varies from 8.3% (68 mmol/mol) to 13.8% (127 mmol/mol). Higher CV of *k* noted in our study in comparison with the study concerning healthy volunteers [12] suggests that the heterogeneity of the life span of the erythrocytes might be higher in patients with diabetes. This is in line with the results reported by Virtue *et al.* [25], who demonstrated that in a group of patients with type 2 diabetes, this parameter was equal to 25 days.

Higher CV noted in our study suggests that the other above-mentioned sources of variability might also be more pronounced or that *k* is more significantly influenced by other factors (e.g., oxidative stress [26]) in patients with diabetes than in healthy subjects. Further studies are required to confirm this hypothesis. Nevertheless, the model also can be individualized, applying the method used in this study, to fit a particular patient’s data more precisely.

The HbA_1c_ model with *k* individually identified for each patient was used to predict HbA_1c_ in cultures of patients’ erythrocytes. We tested a few possible modes of erythrocyte removal, because of a lack of any method that could actually measure which erythrocytes are lost. The results indicated a high ability of the model to predict HbA_1c_ when two modes of erythrocyte removal – chronological loss and uniform loss – were combined. The results of *in vitro* studies strengthen the validity of the model under *in vivo* conditions, because both models share the same *k* for a particular subject and the *in vitro* simulation starts with the calculated HbA_1c_ levels in each equal-aged cohort of erythrocytes on the last day of the glucose monitoring *in vivo*. This means that the two models are strongly interrelated [7].

The average linear relationship of HbA_1c_ as a function of MBG, which was modeled using the mean *k* and a constant 120-day life span of erythrocytes, reproduced the relationship of these variables obtained using the data sampled from the ADAG population despite differences between the groups (e.g., in terms of ethnicity, proportion of nondiabetic individuals and patients with type 1 and type 2 diabetes). This is yet another confirmation of the validity of the model.

More importantly, the fact that both linear relationships are almost identical has significant implications regarding the glycation rate, the life span of erythrocytes, and the glycemic control. On the one hand, if the life span of erythrocytes shortens with a worsening of glycemic control, as was suggested in a few reports [25,27], then the mean *k* must increase to compensate for the shorter time of glycation (otherwise the slopes of both lines would have to be different). Possible mechanisms responsible for such an increase include patients’ susceptibility to oxidative stress and an association of hyperglycemia with free-radical-mediated lipid peroxidation [26] or the existence of high and low hemoglobin glycation phenotypes [28,29]. On the other hand, some studies have indicated that the life span of erythrocytes is independent of glycemic control or even that it is longer in patients with poorer control [18,30,31], implying that the mean glycation rate is not correlated or that it is negatively correlated with glycemic control.

Unfortunately, based solely on the hemoglobin glycation model, it is not possible to judge, whether the life span of erythrocytes and the glycation rate constant are negatively, positively, or not correlated with HbA_1c_. This is related to the fact that in the model the glycation rate constant *k* and the life span of erythrocytes are not present separately but as a product of these two variables (see, above, the second equation in section *Estimation of the overall glycation rate constant*). Therefore, it is not possible to reach a conclusion about possible changes of one of these parameters as a function of HbA_1c_ without having prior knowledge about changes of the other parameter. A reliable method of measuring the survival of erythrocytes *in vivo* is required to solve this problem. In the absence of such a method, mathematical modeling can be used to incorporate a description of the aging of erythrocytes into the glycation model [32].

From a practical point of view, the most important conclusion, that can be drawn from a good agreement of both linear relationships considered above, is that changes in the life span must be balanced by changes in the glycation rate across the wide range of HbA_1c_ levels to ensure nearly a constant product of these two variables; otherwise it would not be possible to reproduce the average relationship of HbA_1c_ and MBG obtained in the ADAG study using the model with *k* equal to 1.296 × 10^−9^ l mmol^−1^ s^−1^ and the 120-day life span of erythrocytes. In fact, the same result would be achieved even if some other value of the life span of erythrocytes were assumed. This is related to the fact that *k* is estimated from the model based on the assumed life span of erythrocytes, that is, the shorter the life span, the higher the estimated value of *k* and vice versa.

Because of a good agreement of the simulated and the experimental relationship of HbA_1c_ and MBG obtained using the mean *k* and the same mean life span of erythrocytes, the same values of these parameters can be used to obtain reliable predictions of HbA_1c_ in response to different glycemic profiles in the average patient with diabetes. We conducted a few series of such predictions for different glycemic profiles preceding the HbA_1c_ test execution. The conclusions from these predictions are as follows: (1) interpreting HbA_1c_ as a measure of MBG is meaningful only in the case of stable glycemic control; (2) the HbA_1c_ level might vary widely during sudden changes in glycemia, even in the case of glycemia profiles with the same MBG level; (3) HbA_1c_ is not a sensitive indicator of short-term glycemic variability; and (4) there is a considerable ambiguity in interpreting the result of a single HbA_1c_ measurement when no additional information about the patient is available (e.g., previous HbA_1c_ or BG values).

These conclusions are in good agreement with observations known from clinical practice and demonstrated in clinical trials. However, using the model it is possible to assess what quantitative statements (for example, “HbA_1c_ is not a sensitive indicator of the short-term glycemic variability”) really mean in terms of concrete numerical values of HbA_1c_ and BG.

From a practical standpoint, the most important value of the presented work is that having a positively verified model, in which k can be identified for a particular patient, and taking into consideration the relative mathematical simplicity of the model, more frequent tests of HbA_1c_ might be used together with the results of modeling to decrease ambiguity of interpreting HbA_1c_ in terms of glycemic control. For example, one can calculate monthly estimates of MBG using the model and monthly HbA_1c_ tests instead of assuming that BG was constant for the whole life span of erythrocytes. In other words, the model make it possible to improve interpretability of the most recent HbA_1c_ value by combining all the evidence available within 3–4 months related to glycemia monitoring and HbA_1c_ testing.

## Conclusions

Our results support the notion that patients with type 1 and type 2 diabetes are characterized by the same mean value of the overall glycation rate constant. There is no significant difference between the mean value of this parameter in patients with diabetes and in healthy individuals.

The obtained results suggest that reciprocal changes in glycation rate and the life span of erythrocytes exist in a wide range of HbA_1c_ values. Thus, for an average patient with either type 1 or type 2 diabetes, no modifications of parameters of the hemoglobin glycation model are required to obtain meaningful HbA_1c_ predictions. Such a model can be used to simulate the influence of different glycemia courses on HbA_1c_ level, making it possible to go beyond the averaged linear relationship of HbA_1c_ and the mean glucose level over the whole life span of erythrocytes. Simulation experiments that were conducted confirm observations known from the clinical practice (i.e., that interpretation of HbA_1c_ as a measure of MBG is fully justified only in the case of the stable glycemic control and that HbA_1c_ is not a sensitive indicator of short-term glycemic variability).

There is considerable intersubject variation in the relationship of HbA_1c_ and the mean blood glucose level. Based on evidence from the literature, the variation is more likely related to the heterogeneity of the life span of erythrocytes than to variability of the glycation rate constant, but further studies are required to confirm this hypothesis. Nevertheless, the model can be individualized to fit a particular subject’s data by applying the method used in our study. The model and more frequent tests of HbA_1c_ might be used to decrease ambiguity of interpreting HbA_1c_ in terms of glycemic control.

## Abbreviations

ADAG: A1C-Derived Average Glucose Study
BG: Blood glucose concentration
CGM: Continuous glucose monitoring
CV: Coefficient of variation
HbA_1c_: Glycated hemoglobin A_1c_
ID: Method of extrapolating 120-day glycemia course based on the continuous glucose monitoring data without averaging
IFCC: International Federation of Clinical Chemistry and Laboratory Medicine
*k*: Overall hemoglobin glycation rate constant
LS: Life span of erythrocytes
MAD: Mean absolute difference (of the measured and the modelled HbA_1c_ levels)
MBG: Mean blood glucose concentration
MD: Mean difference (of the measured and the modelled HbA_1c_ levels)
NGSP: National Glycohemoglobin Standardization Programme
WW: Method of extrapolating 120-day glycemia course based on two mean daily glucose profiles representing working days and weekends
